# Molecular Surveillance of *Dirofilaria immitis* in Field-Collected *Aedes albopictus* from Southeastern Virginia

**DOI:** 10.3390/pathogens15090906

**Published:** 2026-08-28

**Authors:** Jonathan Karisa, Amrita Ray Mohapatra, Lorelei Sandland, Charles Abadam, Karen Akaratovic, Jay Kiser, Goudarz Molaei

**Affiliations:** 1 Center for Vector Biology & Zoonotic Diseases, Department of Entomology, The Connecticut Agricultural Experiment Station, 123 Huntington Street, New Haven, CT 06511, USA; 2 Molecular Biology and Microbiology, Tufts University School of Medicine, 1365 Harrison Avenue, Boston, MA 02111, USA; 3 Suffolk Mosquito Control, Department of Public Works, 800 Carolina Road, Suffolk, VA 23434, USA; 4 Department of Epidemiology of Microbial Diseases, Yale School of Public Health, 60 College Street, New Haven, CT 06510, USA

**Keywords:** *Dirofilaria immitis*, dirofilariasis, heartworm disease, *Aedes albopictus*, Virginia, United States

## Abstract

*Dirofilaria immitis*, also known as dog heartworm, is a filarial nematode of medical and veterinary importance. It is primarily transmitted by mosquitoes of the genera *Aedes*, *Anopheles*, *Culex*, and *Mansonia*, including the invasive *Aedes albopictus*. While primarily a veterinary pathogen, this parasite occasionally causes cutaneous or pulmonary dirofilariasis in humans. The aim of this study was to investigate the status of *D. immitis* in *Ae. albopictus*, a competent invasive vector, and to assess its prevalence in the mid-Atlantic United States. *Aedes albopictus* specimens were collected from various urban and suburban localities in Suffolk, Virginia, during 2023. Genomic DNA extracted from the mosquitoes was screened for *D. immitis* using diagnostic genes in PCR assays, and the resulting products were sequenced to confirm the presence of this parasite’s DNA. Additionally, the host choice of *Ae. albopictus* was identified using PCR assays targeting the mitochondrial cytochrome *b* gene and sequencing. *Dirofilaria immitis* was detected in 9.2% (53/579) of the tested mosquito specimens, comprising 12.1% (46/379) of blood-fed and 3.5% (7/200) of non-blood-fed mosquitoes. *Aedes albopictus* predominantly obtained their blood meals from domestic cats and Virginia opossums. Among the *D. immitis*-positive specimens, the majority had obtained their blood meals from domestic cats and white-tailed deer. Our study provides evidence of the potential role of *Ae. albopictus* in the maintenance and transmission of *D. immitis* in this region and highlights the utility of xenosurveillance for tracking pathogen circulation in animal populations.

## 1. Introduction

Filarial parasites pose an under-recognized risk to the health of domesticated animals and wildlife worldwide [1]. Domesticated dogs serve as the primary hosts for various filarial worm species, including *Dirofilaria immitis*, the causative agent of dirofilariasis—commonly known as heartworm disease. This parasite is also capable of causing dirofilariasis in several other canine and feline species, as well as in other mammalian species, including humans [2], although felines and humans are generally considered incidental hosts [3]. As a mosquito-borne zoonotic filarial nematode, *D. immitis* has emerged as a significant concern globally, including in the United States. Although this endemic parasite has been reported from 50 states, transmission is most prevalent in the southeastern and south-central regions, with state-level incidence ranging from 0.27 to 7.78% in 2018 [2].

The life cycle of *D. immitis* involves an invertebrate vector (a mosquito) and a definitive vertebrate host (usually a canine). During a blood meal from an infected vertebrate host, mosquitoes acquire microfilariae nematodes, which undergo a series of complex developmental stages within the mosquito. This process culminates in the formation of infective third-stage larvae (L3), which are then transmitted to vertebrate hosts during subsequent blood meals [4,5,6]. An increase in *D. immitis* infections in definitive hosts indicates a higher potential for spillover to humans [7]. Although human infections are typically occasional and asymptomatic, they can result in critical outcomes if the parasite infects the pulmonary arteries or the right ventricle [4,8,9].

*Dirofilaria immitis* can be transmitted by several mosquito species across multiple genera, including *Aedes*, *Anopheles*, *Armigeres*, *Coquillettidia*, *Culex*, *Mansonia*, and *Psorophora* [10]. However, not all mosquito species within these genera are effective vectors; there is significant variation in their vector competency [11]. Most recently, *D. immitis* has been detected in the heads of *Aedes aegypti* and *Ae. albopictus*. This suggests these species could play a critical role in transmitting the parasite to canines and spillover into the human population, driven by their mammophilic and anthropophilic feeding behaviors [12,13,14].

*Aedes albopictus* has rapidly spread across the United States since its introduction in 1987. Despite its wide distribution and recognized role in the transmission of arboviruses to humans, its significance as a vector for other pathogens, such as *D. immitis,* remains poorly understood. Determining how *Ae. albopictus* contributes to the transmission dynamics of *D. immitis* could provide valuable insights into the parasite’s geographic distribution. Furthermore, this information may help track its regional spread and clarify the public health risks associated with *D. immitis* infections. Consequently, this study aimed to detect *D. immitis* within *Ae. albopictus* populations in Suffolk, Virginia.

## 2. Materials and Methods

### 2.1. Mosquito Collection

Mosquito sampling was performed weekly from May to November in Suffolk, Virginia, following the protocol described earlier [15]. Sampling was conducted using Centers for Disease Control and Prevention (CDC) miniature light traps (BioQuip Products, Rancho Dominguez, CA, USA) baited with carbon dioxide (CO_2_; compressed gas cylinder; dispersal rate 200 mL/min), Biogents Sentinel traps (Biogents, Regensburg, Germany) baited with both a chemical lure and CO_2_, and modified Reiter gravid traps baited with a fermented mixture of chicken manure, alfalfa, yeast, and water. Most traps were situated in developed residential or commercial areas adjacent to swamps and woodland habitats. Upon collection, traps were transported to the Suffolk Mosquito Control laboratory, where mosquitoes were immobilized in a −18 °C freezer and identified to species level using an identification guide [16] and a dissecting microscope. Specimens were individually placed in 2.0 mL microcentrifuge tubes, labeled, and stored at −80 °C pending further analysis.

### 2.2. Molecular Analysis

Deoxyribonucleic acid (DNA) was isolated from individual whole mosquitoes using DNAzol BD (Molecular Research Center, Cincinnati, OH, USA) in accordance with the manufacturer’s recommendations, with modifications as previously described [17,18]. Briefly, individual mosquitoes were homogenized in 500 µL of DNAzol BD. Following the addition of 15 µL of proteinase K solution, the homogenate was incubated at 70 °C for 10 min and then centrifuged at 15,000 rpm for 10 min. To the resulting supernatant, 200 µL of absolute ethanol and 3 µL of PolyAcryl carrier (Molecular Research Center) were added; the mixture was centrifuged at 10,000 rpm for 10 min to precipitate DNA. After discarding the supernatant, the DNA pellet was washed twice with 750 µL of 75% ethanol and air-dried for 4 min. Finally, DNA was reconstituted in 30 µL of 1X Tris-EDTA buffer, incubated at 65 °C for 10 min, and stored at −20 °C for further analysis.

For the detection of *D. immitis*, the extracted genomic DNA was used as a template to amplify portions of the 16S ribosomal RNA (rRNA) and the cytochrome oxidase subunit 1 (*COI*) genes (Table 1) using single-plex assays [3]. Assays were performed in a hierarchical manner. First, DNA was amplified using 16S rRNA (pan-filarial) primers, and positive samples were subsequently analyzed using COI (species-specific) primers.

Each 40 μL reaction mixture contained 3 µL of template DNA, 3.2 µL of each primer (at a concentration of 5 µM), 4 μL of 10X Qiagen PCR buffer (containing 15 mM MgCl_2_), 0.8 µL of dNTP mix (10 mM each), 0.2 µL of Taq DNA Polymerase (1.25 U/reaction) (Qiagen Sciences Inc., Germantown, MD, USA), and 25.6 µL of nuclease-free water. All amplifications were performed using a GeneAmp PCR System 9700 (Applied Biosystems, Foster City, CA, USA) under the following thermal cycling conditions: an initial denaturation at 94 °C for 2 min; 32 cycles of 94 °C for 30 s, annealing at 58 °C for 1 min (COI species-specific primers) or 60 °C for 1 min (16S rRNA universal primers), and extension at 72 °C for 30 s; followed by a final extension at 72 °C for 7 min. Positive and negative controls were included in every run. Amplicons (10 µL) were electrophoresed on a 2% agarose gel, stained with SmartGlow Pre-stain Solution (Accuris Instruments, Edison, NJ, USA), and visualized under ultraviolet light using a GelDoc system (UVP, Upland, CA, USA). Successful PCR products were purified using QIAquick PCR purification kit (Qiagen), and bidirectional sequencing was conducted at the Keck Sequencing Facility (Yale University, New Haven, CT, USA) using a 3730xl DNA Analyzer (Applied Biosystems). Sequences were annotated with ChromasPro, version 1.22 (Technelysium Pty Ltd., Tewantin, Australia) and compared against the NCBI reference database using the nucleotide Basic Local Alignment Search Tool (BLASTn; https://blast.ncbi.nlm.nih.gov/ (Accessed on 17 April 2025)).

To determine the feeding patterns of *Ae. albopictus*, blood meal analysis was performed by subjecting DNA extracted from blood-fed mosquitoes to PCR amplification using primers targeting the mammalian and avian mitochondrial cytochrome *b* gene (*cyt b*) (Table 1), as previously described [18]. The PCR reaction mixture consisted of 3.2 µL of each primer (at a concentration of 0.5 µM), 2.5 μL of 10X Qiagen PCR buffer (containing 15 mM MgCl_2_), 0.5 µL of dNTP mix (10 mM each), 0.125 µL of Taq DNA Polymerase (1.25 U/reaction), and 27.475 µL of water, and 3 µL of template DNA, for a total reaction volume of 40 μL. Thermal cycling conditions for the mammalian PCR included an initial denaturation at 95 °C for 10 min, followed by 36 cycles of denaturation at 94 °C for 30 s, annealing at 55 °C for 45 s, and extension at 72 °C for 90 s, with a final extension at 72 °C for 7 min. The avian conditions consisted of 95 °C for 5 min, followed by 36 cycles of denaturing at 94 °C for 30 s, annealing at 60 °C for 50 s, and extension at 72 °C for 40 s, with a final extension at 72 °C for 7 min. Both positive and negative controls were included in all reactions. A 10 µL aliquot of each amplification product was electrophoresed on 1.2% agarose gel, stained with SmartGlow Pre-stain Solution (Accuris Instruments), visualized under ultraviolet light, and documented using a GelDoc system (UVP). The resulting amplicons were purified and sequenced as described above. BLASTn was used to compare the generated sequences to the NCBI reference DNA sequences database; sequences with >95% identity were used for host identification.

### 2.3. Data Analysis

Data were managed in Microsoft Excel (Microsoft Corp., Redmond, WA, USA) and analyzed using R version 4.5.2 within RStudio. Descriptive statistics were employed to summarize the proportions of *D. immitis* positivity categorized by blood meal host.

## 3. Results

A total of 2546 *Ae. albopictus* were collected for this study, comprising 22.5% (*n* = 574) blood-fed and 77.5% (*n* = 1972) non-blood-fed individuals. Of these, 579 were screened for the presence of *D. immitis*, including 65.5% (*n* = 379) blood-fed and 34.5% (*n* = 200) non-blood-fed mosquitoes. *Dirofilaria immitis* was detected in 9.2% (*n* = 53) of the tested specimens, consisting of 12.1% (46/379) blood-fed and 3.5% (*n* = 7) non-blood-fed individuals. A subsample of the annotated sequence data that tested positive for *D. immitis* was deposited in the National Center for Biotechnology Information GenBank under Accession Nos. PQ499120, PQ499449, PQ499505, PQ522522, and PQ522526.

A total of 124 samples, comprising 96 negatives and 28 positives for *D. immitis*, were screened for blood meal sources. Among the *D. immitis*-positive specimens of *Ae. albopictus*, the most frequent blood meals were from domestic cats (*n* = 16), followed by white-tailed deer (*n* = 2). Ten samples failed to amplify, likely due to advanced mosquito blood meal digestion stage, low initial blood volume leading to insufficient template DNA, the presence of hemoglobin degradation products and other naturally occurring blood compounds that inhibit Taq DNA Polymerase, or inadequate sample storage (e.g., delayed freezing at −20 °C or −80 °C, thawing during transport, or repeated freeze and thaw cycles) [21,22,23,24]. Similarly, most of the *D. immitis*-negative specimens obtained blood meals from domestic cats (*n* = 44), followed by Virginia opossums (*n* = 17), common box turtles (*n* = 4), humans (*n* = 3), black rats and white-tailed deer (*n* = 2 each), and dogs and cottontail rabbits (*n* = 1 each). The remaining samples either failed to amplify in PCR assays or yielded sequencing results below the 95% identity threshold (Table 2). None of the *Ae. albopictus* had obtained blood meals from birds.

## 4. Discussion

This study provides insights into the potential role of *Ae. albopictus* in transmission of *D. immitis* in Suffolk, Virginia. A comprehensive understanding of the parasite prevalence within mosquito populations is essential for designing targeted control measures. Our findings indicate that 9.2% (53/579) of the tested *Ae. albopictus* were positive for *D. immitis* parasites.

In the present study, *D. immitis* was detected in 9.2% of the tested specimens, comprising 12.1% blood-fed and 3.5% non-blood-fed individuals. A recent study in the Lower Rio Grande Valley in south Texas reported that 21% of blood-fed *Ae. aegypti* with blood meals originating from dogs tested positive for *D. immitis*, while 7% of those that fed on humans tested positive [13]. In another molecular survey in the United States, parasite DNA was detected in 6.3% of dogs from 17 of 39 states and in 0.3% of the cats across 4 of 42 states [2]. Consistently, an earlier investigation of a comprehensive canine infection database found that *D. immitis* antigen was most commonly identified in the South (3.9%) [25]. Furthermore, an analysis of over 144 million test results from 2013 to 2019 identified substantial regional variations: *D. immitis* antibody prevalence ranged from 0.4% to 2.9% across 50 states, with the highest detection rates in the Southeast (2.6%) [26].

Studies have also identified other mosquito species, including *Anopheles crucians* and *Anopheles punctipennis*, as positive for *D. immitis* [9,27]. Our detection of the parasite in the non-blood-fed *Ae. albopictus* suggests two possibilities: residual parasite DNA from a prior blood meal or early-stage development within the abdomen before the infective L3 stage migrates to the proboscis. Consequently, the role of *Ae. albopictus* as a potential vector warrants serious consideration. Furthermore, an evaluation of long-term mosquito surveillance and dog heartworm case data in California via statistical modeling revealed significant regionally specific associations between heartworm cases and the abundance of four vector species: *Ae. aegypti* (Central California), *Ae. albopictus* (Southern California), *Aedes sierrensis* (Central California), and *Culiseta incidens* (Northern and Central California) [28].

Although limited in sample size, the present study observed that, *Ae. albopictus* fed on mammalian species—including domestic cats, Virginia opossums, humans, black rats, dogs, cottontail rabbits, and white-tailed deer—as well as one reptilian species, the common box turtle. Similarly, a recent investigation of the blood-feeding behavior of 961 engorged specimens of *Ae. albopictus* collected from the same region (Suffolk, Virginia) reported domestic cats as the most frequent host (50.5%), followed by Virginia opossums (17.1%), white-tailed deer (12.2%), and humans (7.3%); together representing 87.1% of all identified blood meals [15]. An earlier study in New Jersey also found that *Ae. albopictus* fed on humans, cats, dogs, and Virginia opossums, noting that human-derived blood meals were significantly more frequent in suburban areas, whereas cat-derived blood meals were more prevalent in urban habitats [29]. In Suffolk, where this study was conducted, domestic and feral cats are common in urban areas where engorged *Ae. albopictus* were collected. Feral cat populations in low-income urban neighborhoods are largely unmanaged by local animal control agencies (aside from sporadic trapping), creating high host availability. These free-ranging, nocturnal cats frequently rest in shaded vegetation during the day—a habitat that directly aligns with the diurnal resting and opportunistic host-seeking behavior of *Ae. albopictus*, likely contributing to the high proportion of blood meals taken from feline hosts [30].

Conversely, only a single dog blood meal was identified in our samples (which tested negative for *D. immitis*). This low representation may reflect lower relative physical accessibility of dogs during peak *Ae. albopictus* feeding hours (e.g., dogs kept indoors or in cleared yard areas with less shaded vector habitat) as well as defensive behaviors that disrupt mosquito feeding [31].

Regarding the high proportion of *D. immitis*-positive blood meals derived from cats, two potential mechanisms must be considered. First, while cats are generally considered atypical or dead-end hosts for *D. immitis*—frequently exhibiting low, transient, or amicrofilaremic infections—a subset of infected, untreated free-ranging cats can develop microfilaremia sufficient to infect feeding mosquitoes. Second, positive PCR detection in cat-derived blood meals may represent “dead-end ingestion,” wherein mosquitoes acquire microfilariae or L3-stage larvae during blood feeding without the host necessarily serving as an effective transmission reservoir. Given the absence of routine veterinary care and heartworm prophylaxis among feral populations in these areas, free-ranging cats may play a more active role in the local transmission dynamics of *D. immitis* than previously recognized, warranting further investigation into feline microfilaria loads in urban settings [32,33].

Our study provides further evidence for the utility of xenosurveillance in tracking pathogen circulation within animal populations. By analyzing blood-fed *Ae. albopictus*, we simultaneously identified both vertebrate hosts and the pathogens they harbored. Xenosurveillance has been previously employed to detect *Loa loa*, *Wuchereria bancrofti*, *Mansonella perstans*, and *Plasmodium falciparum* [34], dengue virus [35], West Nile virus [36], and Rift Valley fever virus [37]. This approach offers a cost-effective, unbiased, and scalable solution for pathogen surveillance, particularly for elucidating hidden transmission cycles in reservoirs difficult to sample.

## 5. Study Limitations

This study was limited by logistical challenges, which restricted our analysis to a subset of samples for both *D. immitis* DNA detection and blood meal identification. Although these data provide insights into the presence of *D. immitis* in Suffolk, Virginia, further research is warranted to fully elucidate the distribution and role of *Ae. albopictus* in the transmission of this parasite.

## 6. Conclusions

Our study detected *D. immitis* DNA in both non-blood-fed and blood-fed *Ae. albopictus*. The latter had obtained blood meals from domestic cats and white-tailed deer, suggesting that this invasive mosquito species plays a role in the transmission and maintenance of the filarial parasite in this region of the mid-Atlantic USA. Furthermore, our study also provides evidence for the utility of xenosurveillance in tracking pathogen circulation in animal populations.

## Figures and Tables

**Table 1:** Sequences of primers, target genes, and amplicon sizes used for *Dirofilaria immitis* detection and blood meal identification.

Assay	Oligo Name	Oligonucleotide Sequence (5′–3′)	Gene Targeted	Amplicon Size (bp)	Reference
*D. immitis* detection					
	DIDR-F1	AGTGCGAATTGCAGACGCATTGAG	*16S rRNA*	430–664	[19]
	DIDR-R1	AGCGGGTAATCACGACTGAGTTGA			
	DI-COI-F1	AGTGTAGAGGGTCAGCCTGAGTTA	*COI*	203	[19]
	DI-COI-R1	ACAGGCACTGACAATACCAAT			
Blood meal identification					
	MamF	CGAAGCTTGATATGAAAAACCATCGTTG	*cyt b* *cyt b*	772	[20]
	MamR	TGTAGTTRTCWGGGTCHCCTA			
	AVIF	GACTGTGACAAAATCCCNTTCCA		508	[20]
	AVIR	GGTCTTCATCTYHGGYTTACAAGAC			

**Table 2:** Mammalian and reptilian hosts of *Aedes albopictus* mosquitoes and *Dirofilaria immitis* detection.

Host	Common Name	Negative	Positive
*Canis lupus familiaris*	Dog	1	0
*Didelphis virginiana*	Virginia opossum	17	0
*Felis catus*	Domestic cat	44	16
*Homo sapiens*	Human	3	0
*Odocoileus virginianus*	White-tailed deer	2	2
*Rattus rattus*	Black rat	2	0
*Sylvilagus* spp.	Cottontail rabbit	1	0
*Terrapene carolina*	Common box turtle	4	0
Unamplified	N/A	22	10
Total		96	28

## Data Availability

The datasets supporting the findings may be provided by the corresponding author upon request.

## References

[1] Dantas-Torres F., Otranto D. (2020). Overview on *Dirofilaria immitis* in the Americas, with notes on other filarial worms infecting dogs. Vet. Parasitol..

[2] Smith R., Murillo D.F.B., Chenoweth K., Barua S., Kelly P.J., Starkey L., Blagburn B., Wood T., Wang C. (2022). Nationwide molecular survey of *Dirofilaria immitis* and *Dirofilaria repens* in companion dogs and cats, United States of America. Parasit. Vectors.

[3] Torres-Chable O.M., Baak-Baak C.M., Cigarroa-Toledo N., Blitvich B.J., Brito-Argaez L.G., Alvarado-Kantun Y.N., Zaragoza-Vera C.V., Arjona-Jimenez G., Moreno-Perez L.G., Medina-Perez P. (2018). Molecular detection of *Dirofilaria immitis* in dogs and mosquitoes in Tabasco, Mexico. J. Vector Borne Dis..

[4] Geary J., Satti M., Moreno Y., Madrill N., Whitten D., Headley S.A., Agnew D., Geary T., Mackenzie C. (2012). First analysis of the secretome of the canine heartworm, *Dirofilaria immitis*. Parasit. Vectors.

[5] Huang S., Smith D.J., Molaei G., Andreadis T.G., Larsen S.E., Lucchesi E.F. (2013). Prevalence of *Dirofilaria immitis* (Spirurida: Onchocercidae) infection in *Aedes*, *Culex*, and *Culiseta* mosquitoes from north San Joaquin Valley, CA. J. Med. Entomol..

[6] McCrea A.R., Edgerton E.B., Oliver G.T., O’Neill F.M., Nolan T.J., Lok J.B., Povelones M. (2021). A novel assay to isolate and quantify third-stage *Dirofilaria immitis* and *Brugia malayi* larvae emerging from individual *Aedes aegypti*. Parasit. Vectors.

[7] Drake J., Wiseman S. (2018). Increasing incidence of *Dirofilaria immitis* in dogs in USA with focus on the southeast region 2013–2016. Parasit. Vectors.

[8] Bowman D.D., Liu Y., McMahan C.S., Nordone S.K., Yabsley M.J., Lund R.B. (2016). Forecasting United States heartworm *Dirofilaria immitis* prevalence in dogs. Parasit. Vectors.

[9] Licitra B., Chambers E.W., Kelly R., Burkot T.R. (2010). Detection of *Dirofilaria immitis* (Nematoda: Filarioidea) by polymerase chain reaction in *Aedes albopictus*, *Anopheles punctipennis*, and *Anopheles crucians* (Diptera: Culicidae) from Georgia, USA. J. Med. Entomol..

[10] Younes L., Barré-Cardi H., Bedjaoui S., Ayhan N., Varloud M., Mediannikov O., Otranto D., Davoust B. (2021). *Dirofilaria immitis* and *Dirofilaria repens* in mosquitoes from Corsica Island, France. Parasit. Vectors.

[11] Ferreira C.A.C., De Pinho Mixão V., Novo M.T.L.M., Calado M.M.P., Gonçalves L.A.P., Belo S.M.D., De Almeida A.P.G. (2015). First molecular identification of mosquito vectors of *Dirofilaria immitis* in continental Portugal. Parasit. Vectors.

[12] Ledesma N.A., Kaufman P.E., Xue R.-D., Leyen C., Macapagal M.J., Winokur O.C., Harrington L.C. (2019). Entomological and sociobehavioral components of heartworm (*Dirofilaria immitis*) infection in two Florida communities with a high or low prevalence of dogs with heartworm infection. J. Am. Vet. Med. Assoc..

[13] Scavo N.A., Zecca I.B., Sobotyk C., Saleh M.N., Lane S.K., Olson M.F., Hamer S.A., Verocai G.G., Hamer G.L. (2022). High prevalence of canine heartworm, *Dirofilaria immitis*, in pet dogs in south Texas, USA, with evidence of *Aedes aegypti* mosquitoes contributing to transmission. Parasit. Vectors.

[14] Vezzani D., Mesplet M., Eiras D.F., Fontanarrosa M.F., Schnittger L. (2011). PCR detection of *Dirofilaria immitis* in *Aedes aegypti* and *Culex pipiens* from urban temperate Argentina. Parasitol. Res..

[15] Little E., Harriott O.T., Akaratovic K.I., Kiser J.P., Abadam C.F., Shepard J.J., Molaei G. (2021). Host interactions of *Aedes albopictus*, an invasive vector of arboviruses, in Virginia, USA. PLoS Negl. Trop. Dis..

[16] Harrison B.A., Byrd B.D., Sither C.B., Whitt P.B. (2016). The Mosquitoes of the Mid-Atlantic Region: An Identification Guide.

[17] Khalil N., Little E.A.H., Akaratovic K.I., Kiser J.P., Abadam C.F., Yuan K.J., Misencik M.J., Armstrong P.M., Molaei G. (2021). Host Associations of *Culex pipiens*: A two-year analysis of bloodmeal sources and implications for arboviral transmission in Southeastern Virginia. Vector Borne Zoonotic Dis..

[18] Molaei G., Oliver J., Andreadis T.G., Armstrong P.M., Howard J.J. (2006). Molecular identification of blood-meal sources in *Culiseta melanura* and *Culiseta morsitans* from an endemic focus of eastern equine encephalitis virus in New York. Am. J. Trop. Med. Hyg..

[19] Rishniw M., Barr S.C., Simpson K.W., Frongillo M.F., Franz M., Alpizar J.L.D. (2006). Discrimination between six species of canine microfilariae by a single polymerase chain reaction. Vet. Parasitol..

[20] Ngo K.A., Kramer L.D. (2003). Identification of mosquito blood meals using polymerase chain reaction (PCR) with order-specific primers. J. Med. Entomol..

[21] Oshaghi M.A., Chavshin A.R., Vatandoost H., Yaaghoobi F., Mohtarami F., Noorjah N. (2006). Effects of post ingestion and physical conditions on PCR amplification of host blood meal DNA in mosquitoes. Exp. Parasites.

[22] Molaei G., Andreadis T.G., Armstrong P.M., Diuk-Wasser M. (2008). Host-feeding patterns of potential mosquito vectors in Connecticut, USA: Molecular analysis of bloodmeals from 23 species of *Aedes*, *Anopheles*, *Culex*, *Coquillettidia*, *Psorophora*, and *Uranotaenia*. J. Med. Ent..

[23] Molaei G., Cummings R.F., Su T., Armstrong P.M., Williams G.A., Cheng M.L., Webb J.P., Andreadis T.G. (2010). Vector-host interactions governing epidemiology of West Nile virus in Southern California. Am. J. Trop. Med. Hyg..

[24] Reeves L.E., Holderman C.J., Gillett-Kaufman J.L., Kawahara A.Y., Kaufman P.E. (2016). Maintenance of host DNA integrity in field-preserved mosquito (Diptera: Culicidae) blood meals for identification by DNA barcoding. Parasit. Vectors.

[25] Bowman D., Little S.E., Lorentzen L., Shields J., Sullivan M.P., Carlin E.P. (2009). Prevalence and geographic distribution of *Dirofilaria immitis*, *Borrelia burgdorferi*, *Ehrlichia canis*, and *Anaplasma phagocytophilum* in dogs in the United States: Results of a national clinic-based serologic survey. Vet. Parasitol..

[26] Little S., Braff J., Place J., Buch J., Dewage B.G., Knupp A., Beall M. (2021). Canine infection with *Dirofilaria immitis*, *Borrelia burgdorferi*, *Anaplasma* spp., and *Ehrlichia* spp. in the United States, 2013–2019. Parasit. Vectors.

[27] Nayar J.K., Knight J.W. (1999). *Aedes albopictus* (Diptera: Culicidae): An experimental and natural host of *Dirofilaria immitis* (Filarioidea: Onchocercidae) in Florida, USA. J. Med. Entomol..

[28] Couper L.I., Mordecai E.A. (2022). Ecological drivers of dog heartworm transmission in California. Parasit. Vectors.

[29] Faraji A., Egizi A., Fonseca D.M., Unlu I., Crepeau T., Healy S.P., Gaugler R. (2014). Comparative host feeding patterns of the Asian tiger mosquito, *Aedes albopictus*, in urban and suburban Northeastern USA and implications for disease transmission. PLoS Negl. Trop. Dis..

[30] Richards S.L., Ponnusamy L., Unnasch T.R., Hassan H.K., Apperson C.S. (2006). Host-feeding patterns of *Aedes albopictus* (Diptera: Culicidae) in relation to availability of human and domestic animals in suburban landscapes of central North Carolina. J. Med. Entomol..

[31] Edman J.D., Webber L.A., Schmid A.A. (1987). Host defensive behaviour and the feeding success of mosquitoes. Int. J. Trop. Insect Sci..

[32] McCall J.W., Genchi C., Kramer L.H., Guerrero J., Vencoet L. (2008). Heartworm disease in animals and humans. Adv. Parasitol..

[33] Venco L., Marchesotti M., Manzocchi S. (2015). Feline heartworm disease: A ‘Rubik’s-cube-like’ diagnostic and therapeutic challenge. J. Vet. Cardiol..

[34] Pryce J., Pilotte N., Menze B., Sirois A.R., Zulch M., Agbor J.P., Williams S.A., Wondji C.S., Reimer L. (2022). Integrated xenosurveillance of *Loa loa*, *Wuchereria bancrofti*, *Mansonella perstans* and *Plasmodium falciparum* using mosquito carcasses and faeces: A pilot study in Cameroon. PLoS Negl. Trop. Dis..

[35] Osalla J., Gouagna L.-C., Rotich G., Nzilani M., Safari P., Senagi K., Mutuku F., Torto B., Tchouassi D.P. (2025). Longitudinal surveillance of *Aedes aegypti* (Diptera: Culicidae) in urban coastal Kenya: Population dynamics, blood feeding frequency and dengue virus infection rates. Sci. Rep..

[36] Ndione M.H.D., Ndiaye E.H., Dieng M., Diouf B., Sankhé S., Diallo D., Kane M., Sene N.M., Mbanne M., Sy F.A. (2025). Mosquito-based detection of retroviruses and arboviruses in Senegal: Expanding the scope of xenosurveillance. One Health Outlook.

[37] Lutomiah J., Omondi D., Masiga D., Mutai C., Mireji P.O., Ongus J., Linthicum K.J., Sang R. (2014). Blood meal analysis and virus detection in blood-fed mosquitoes collected during the 2006–2007 Rift Valley fever outbreak in Kenya. Vector Borne Zoonotic Dis..

